# Arritmias Cardíacas em Pacientes com COVID-19

**DOI:** 10.36660/abc.20200963

**Published:** 2021-09-03

**Authors:** Mauricio Pimentel, Ana Paula Arbo Magalhães, Camila Valvassori Novak, Bruna Miers May, Luiz Gustavo Bravosi da Rosa, Leandro Ioschpe Zimerman

**Affiliations:** 1 Hospital de Clinicas de Porto Alegre Porto Alegre RS Brasil Hospital de Clinicas de Porto Alegre, Porto Alegre, RS - Brasil; 2 Universidade Federal do Rio Grande do Sul Cardiologia e Ciências Cardiovasculares Porto Alegre RS Brasil Programa de Pós-Graduação em Ciências da Saúde: Cardiologia e Ciências Cardiovasculares – Universidade Federal do Rio Grande do Sul, Porto Alegre, RS – Brasil

**Keywords:** COVID-19, Arritmias Cardíacas, Parada Cardiorrespiratória, Fibrilação Atrial

## Abstract

**Fundamento:**

A doença pelo novo coronavírus (COVID-19) está associada a manifestações clínicas cardiovasculares, incluindo a ocorrência de arritmias cardíacas.

**Objetivos:**

Avaliar a incidência de arritmias cardíacas (taquiarritmia atrial, bradiarritmia e taquicardia ventricular sustentada) e de parada cardiorrespiratória (PCR) em uma coorte de pacientes internados com COVID-19 em hospital universitário terciário.

**Métodos:**

Estudo de coorte retrospectivo realizado por meio de revisão dos registros de prontuário médico. Para comparação entre os grupos, foi considerado como estatisticamente significativo valor de P < 0,05.

**Resultados:**

Foram incluídos 241 pacientes consecutivos com diagnóstico de COVID-19 (idade média, 57,8 ± 15,0 anos; 51,5% homens; 80,5% de raça branca) e 35,3% com necessidade de ventilação mecânica invasiva (VM). A mortalidade geral foi de 26,6%, sendo de 58,8% entre aqueles em VM. Arritmias cardíacas ocorreram em 8,7% dos pacientes, sendo a mais comum taquiarritmia atrial (76,2%). Pacientes com arritmias apresentaram maior mortalidade, 52,4% versus 24,1% (p=0,005). Em análise multivariada, apenas a presença de insuficiência cardíaca foi associada a maior risco de arritmias ( *hazard ratio* , 11,9; IC 95%: 3,6-39,5; p<0,001). Durante a internação, 3,3% dos pacientes foram atendidos em PCR, com predomínio de ritmos não chocáveis. Todos os atendidos em PCR evoluíram com óbito durante a internação.

**Conclusão:**

A incidência de arritmias cardíacas em pacientes internados com COVID-19 em hospital terciário brasileiro foi de 8,7%, sendo a mais comum taquiarritmias atrial. A presença de insuficiência cardíaca foi associada a maior risco de arritmias. Pacientes com COVID-19 atendidos em PCR apresentam elevada mortalidade.

## Introdução

A doença causada pelo coronavírus da síndrome respiratória aguda grave 2 (SARS-CoV-2), denominada COVID-19, teve seus primeiros casos reportados na China e, em decorrência de sua rápida disseminação mundial, foi declarada pandemia pela Organização Mundial da Saúde em 11 de março de 2020. No Brasil, o número de casos confirmados de COVID-19 ultrapassou 3 milhões em agosto de 2020. ^1^

Nas descrições das séries de casos de COVID-19, foram reportadas complicações cardiovasculares, incluindo injúria miocárdica, arritmias, miocardite, insuficiência cardíaca e choque cardiogênico. ^2^ O dano ao sistema cardiovascular provavelmente tem causa multifatorial, incluindo lesão cardíaca direta pelo vírus, resposta inflamatória sistêmica exacerbada e fenômenos tromboembólicos. ^3^ A ação do vírus por meio do receptor da enzima conversora de angotensina 2 e seu efeito de *down regulation* são fatores envolvidos na resposta inflamatória exacerbada. ^4^ No caso das arritmias cardíacas, pode-se considerar também a possibilidade de efeitos pró-arrítmicos de drogas utilizadas para tratamento da COVID-19, hipóxia causada por envolvimento viral pulmonar, isquemia miocárdica, distúrbios hidroeletrolíticos, “ *strain* ” miocárdico e alterações de volume intravascular. ^5^ A resposta inflamatória desbalanceada por células T *helper* dos tipos 1 e 2 constitui ainda outro mecanismo proposto para explicar inflamação e arritmogênese em pacientes com COVID-19. ^6^ As primeiras séries de casos da China mostraram incidência de arritmias cardíacas de 17%, podendo chegar a 44% em pacientes internados em unidade de terapia intensiva (UTI). ^7^ Esses estudos, porém, não descreveram de modo detalhado o tipo e as características das arritmias apresentadas. Trabalhos mais recentes em centros norte-americanos apontam incidência geral de arritmias de 6% e de taquiarritmias atriais de 16%. ^8^^–^^10^

Este trabalho tem como objetivo avaliar a incidência de parada cardiorrespiratória e de arritmias cardíacas em uma coorte de pacientes internados com COVID-19 em hospital universitário terciário brasileiro.

## Métodos

Estudo de coorte incluindo pacientes consecutivos internados com diagnóstico de COVID-19 no Hospital de Clínicas de Porto Alegre, Rio Grande do Sul, a partir de 1 de março até 20 de julho de 2020. Foram analisados os primeiros 241 pacientes consecutivos que tiveram diagnóstico confirmado de infecção por SARS-CoV-2 através de RT-PCR de aspirado nasofaríngeo.

Todos os registros de prontuário médico eletrônico foram revisados para obtenção de informações demográficas e comorbidades e ainda de dados sobre desfecho da internação hospitalar (óbito ou alta hospitalar), necessidade de ventilação mecânica invasiva (VM) e ocorrência de parada cardiorrespiratória atendida e de arritmias cardíacas. Foram analisados os registros de evolução médica, de enfermagem e os traçados eletrocardiográficos quando disponíveis no sistema de prontuário eletrônico.

Para os casos de parada cardiorrespiratória, foi revisada informação sobre ritmo inicial, classificado em: fibrilação ventricular/taquicardia ventricular (FV/TV), assistolia, bradiarritmia e atividade elétrica sem pulso (AESP). A ocorrência de arritmias cardíacas foi definida pela presença de taquiarritmias atriais sustentadas (fibrilação atrial, *flutter* atrial), bradiarritmias e taquicardia ventricular sustentada. Não foram incluídas arritmias já presentes na admissão hospitalar, apenas casos incidentes durante a internação. O protocolo do estudo foi aprovado pelo Comitê de Ética em Pesquisa do Grupo de Pesquisa e Pós-Graduação do Hospital de Clínicas de Porto Alegre.

### Análise estatística

As variáveis contínuas com distribuição normal foram descritas através de média e desvio-padrão. O tempo de internação não apresentou distribuição normal pelo teste Shapiro-Wilk, sendo apresentado na forma de mediana e intervalo interquartil e comparado com teste de Mann-Whitney. As características demográficas e clínicas foram comparadas entre pacientes com e sem arritmias cardíacas utilizando-se o teste *t* de Student não pareado para variáveis contínuas e qui-quadrado para variáveis categóricas. A associação entre variáveis clínicas e a ocorrência de arritmias cardíacas foi avaliada com modelos de análise univariada e multivariada de Cox. Foi considerado como estatisticamente significativo um valor de p bicaudal < 0,05. Todas as análises foram realizadas utilizando o programa SPSS, versão 14.0 para Windows.

## Resultados

Neste estudo de coorte foram incluídos 241 pacientes consecutivos internados com COVID-19 e idade média de 57,8 ± 15,0 anos, sendo 51,5% homens e 80,5% de raça branca. A mediana do tempo de internação foi de 9 (intervalo interquartil 5-17) dias, sendo que 35,3% dos pacientes necessitaram de VM. O tempo de internação foi maior naqueles que apresentaram arritmias cardíacas. A mortalidade geral foi de 26,6%, sendo 58,8% entre os que necessitaram de VM e 9% entre os que não necessitaram de VM (p=0,001).

A ocorrência de arritmias cardíacas, definida pela presença de taquiarritmias atriais sustentadas, bradiarritmias e taquicardia ventricular sustentada, foi observada em 21 pacientes (8,7%). A Tabela 1 mostra as características demográficas e clínicas dos pacientes com e sem arritmias. Entre aqueles com arritmias, 16 (76,2%) apresentaram taquiarritmias atriais sustentadas, 3 (9,5%) taquicardia ventricular sustentada e 2 (9,5%) bradiarritmias. Pacientes com arritmias apresentaram maior mortalidade, 52,4% *versus* 24,1% (p=0,005). A ocorrência de arritmias cardíacas foi mais frequente em homens, pacientes em VM e com história de insuficiência cardíaca. A Tabela 2 mostra os resultados da análise univariada e multivariada de Cox para ocorrência de arritmias cardíacas. Nesse modelo, apenas a presença de insuficiência cardíaca foi associada significativamente a maior risco de arritmias cardíacas ( *hazard ratio,* 11,9; IC 95% 3,6-39,5; p<0,001). Em modelo ajustado para a presença de insuficiência cardíaca, a ocorrência de arritmias cardíacas foi associada a maior risco de mortalidade total ( *hazard ratio,* 3,4; IC 95% 1,8-6,7; p<0,05).

**Tabela 1:** Características clínicas dos pacientes com e sem arritmias cardíacas

	Todos os pacientes (n = 241)	Com arritmia (n = 21)	Sem arritmia (n = 220)	Valor p
Idade, anos	57,8 ± 15,0	62,6 ± 13,4	57,3 ± 15,0	0,11
Homens	124 (51,5)	15 (72,4)	109 (49,5)	0,05
IMC, kg/m^2^	30,4 ± 6,3	29,3 ± 5,0	30,5 ± 6,4	0,43
Raça branca	194 (80,5)	17 (81)	177 (80,5)	0,24
Ventilação mecânica	85 (35,3)	14 (66,7)	71 (32,2)	0,002
Internação, dias	9 (5-17)	25 (12-43)	9 (5-16)	0,001
Óbito	64 (26,6)	11 (52,4)	53 (24,1)	0,005
Comorbidades				
HAS	123 (51)	14 (66)	109 (49,5)	0,13
DM	64 (26,6)	7 (33,3)	57 (25,9)	0,46
IC	15 (6,2)	5 (23,8)	10 (4,5)	0,001
Doença pulmonar	52 (21,6)	7 (33,3)	45 (20,5)	0,17
Doença renal crônica	29 (12)	-	29 (13,2)	0,07
Medicações				
Hidroxicloroquina	43 (17,8)	3 (14,3)	40 (18,2)	0,65
Anticoagulantes	39 (16,2)	2 (9,5)	37 (16,8)	0,24

*Dados expressos como média ± desvio-padrão ou números absolutos (percentual). O tempo de internação foi expresso pela mediana e intervalo interquartil. IMC: índice de massa corporal; HAS: hipertensão arterial sistêmica; DM: diabete melito; IC: insuficiência cardíaca* .

**Tabela 2:** Análise univariada e multivariada para o desfecho arritmias cardíacas

	Análise univariada			Análise multivariada		
	HR	IC 95%	p	HR	IC 95%	p
Homens	2,04	0,79-5,32	0,14	1,65	0,62-4,40	0,31
Ventilação mecânica	2,13	0,75-6,04	0,15	2,57	0,88-7,49	0,08
Insuficiência cardíaca	11,10	3,48-35,3	0,01	11,91	3,59-39,46	0,01

*HR: hazard ratio; IC: intervalo de confiança* .

**Figura 1:**
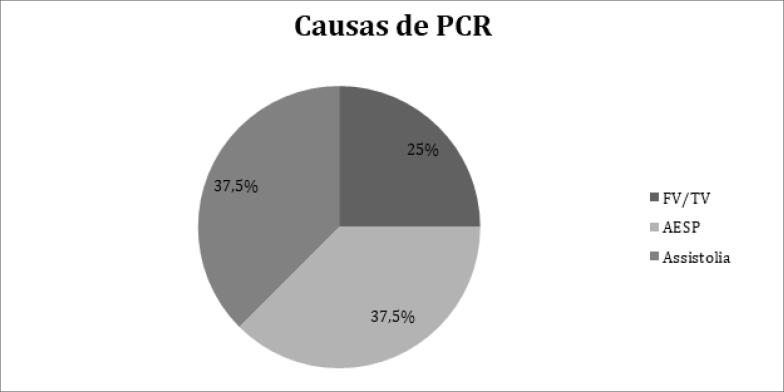
*Ritmos de parada cardiorrespiratória em pacientes com COVID-19. PCR: parada cardiorrespiratória; AESP: atividade elétrica sem pulso; FV/TV: fibrilação ventricular/taquicardia ventricular* .

Durante o período de internação, 8 pacientes (3,3%), todos admitidos em UTI, foram atendidos em parada cardiorrespiratória e suas características clínicas são apresentadas na Tabela 3 . A Figura 1 mostra a distribuição dos ritmos de parada cardiorrespiratória, sendo FV/TV em 2 pacientes (25%), AESP em 3 (37,5%) e assistolia em 3 (37,5%). Todos os pacientes atendidos em parada cardiorrespiratória evoluíram para óbito durante a internação.

**Tabela 3:** Características das paradas cardiorrespiratórias em pacientes com COVID-19

Paciente número	Dia de internação da PCR	Ritmo da PCR	Descrição clínica	Desfecho
1	1	AESP	26 anos, asma, obesidade e esquizofrenia	ROSC 20 minutos, encefalopatia anóxica, instituídas medidas de conforto, óbito
2	26	AESP	54 anos, transplantada renal	ROSC 2 minutos, evolução hospitalar com choque refratário, óbito
3	25	FV/TV	58 anos, miocardiopatia dilatada, portador de CDI, SARA e VM	ROSC 20 minutos, choque refratário, óbito
5	10	FV/TV	45 anos, miocardiopatia dilatada	ROSC 12 minutos, choque refratário, óbito
6	43	Assistolia	71 anos, cardiopatia isquêmica	Óbito
7	12	Assistolia	63 anos, HAS, DM	Óbito
8	01	AESP	76 anos, cardiopatia isquêmica	Óbito
9	25	Assistolia	41 anos, HAS, obesidade	ROSC 35 min, disfunção múltiplos órgãos, óbito

*PCR: parada cardiorrespiratória; AESP: atividade elétrica sem pulso; FV/TV: fibrilação ventricular/taquicardia ventricular; ROSC: retorno da circulação espontânea; CDI: cardioversor desfibrilador implantável; SARA: síndrome da angústia respiratória do adulto; HAS: hipertensão arterial sistêmica; DM: diabete melito; VM: ventilação mecânica* .

## Discussão

Neste estudo de coorte que incluiu pacientes consecutivos internados com COVID-19 em hospital de referência, a mortalidade geral foi de 26,6%, a incidência de arritmias, de 8,7% e a de parada cardiorrespiratória, de 3,3%. A taquiarritmia atrial foi mais comum, correspondendo a 76,2% das arritmias. A presença de insuficiência cardíaca foi a única variável associada a maior risco de arritmias cardíacas em análise multivariada. O tempo de internação de pacientes com arritmias foi maior do que o daqueles sem; isso pode se dever à necessidade de tratamento da própria arritmia ou representar a presença de maior complexidade e gravidade nos casos que desenvolvem arritmias.

As séries iniciais mostraram incidência de arritmias cardíacas em pacientes com COVID-19 variando de 7% a 17%, porém não há descrição específica sobre o seu tipo. ^7^^,^^10^ Na coorte do estado de Nova York, a incidência de arritmias foi associada a diferentes combinações de drogas utilizadas para tratamento da COVID-19, variando de 10% a 20%, porém sem definição dos tipos de arritmia avaliados. ^11^ Em nosso estudo, não encontramos associação do uso de hidroxicloroquina com maior risco de arritmias. A incidência específica de cada tipo de arritmia foi descrita apenas recentemente. Dados de registro internacional que incluiu 1197 profissionais de eletrofisiologia mostram que fibrilação atrial foi a arritmia mais frequentemente descrita em pacientes com COVID-19. ^12^ Em estudo com 115 pacientes, a incidência de taquiarritmias atriais foi de 16,5%, sendo de 27,5% entre os admitidos em UTI. ^8^ No maior estudo específico sobre arritmias publicado até o momento, foram avaliados 700 pacientes hospitalizados por 9 semanas. ^9^ Durante o acompanhamento, 44 deles (6,3%) apresentaram arritmias cardíacas, incluindo fibrilação atrial, bradiarritmias e taquicardia ventricular não sustentada, sendo fibrilação atrial a mais frequente (57%). Presença de insuficiência cardíaca e internação em UTI foram associadas significativamente a maior risco de arritmias. Naquela coorte, 11% dos pacientes foram admitidos em UTI e a mortalidade geral foi de 4%. Nossa incidência de arritmia de 8,7% pode ser considerada próxima à daquele estudo, assim como o predomínio de arritmias atriais e a associação da presença de insuficiência cardíaca com maior risco de arritmias. Por outro lado, em nosso estudo foram incluídos pacientes de maior gravidade (35% com necessidade de VM) e a presença de taquicardia ventricular não sustentada não foi elencada como desfecho. A utilização de VM foi associada à tendência de maior risco de arritmias, porém não foi considerada estatisticamente significativa. A confirmação do achado de que pacientes com insuficiência cardíaca apresentam maior risco de arritmias pode orientar a necessidade de maior monitorização desses pacientes durante internação.

Na pesquisa internacional entre profissionais de eletrofisiologia, 4,8% reportaram casos de FV/TV e 5,6%, de AESP. ^12^ Nas séries iniciais da China, não houve indicação específica sobre a ocorrência de parada cardiorrespiratória e seus ritmos. ^2^^,^^7^ Em um dos trabalhos, a incidência de FV/TV foi de 5,9%, sendo maior entre pacientes com troponina elevada. ^2^ Na coorte do estado de Nova York, a incidência de parada cardiorrespiratória descrita foi de 6% a 15%, variando de acordo com diferentes combinações de drogas utilizadas no tratamento da COVID-19. ^11^ Não foram descritos os ritmos de parada cardiorrespiratória. No estudo de Bhatla *et al* . já descrito acima, foram relatados 9 casos de parada cardiorrespiratória (1,3%), sendo 6 casos de AESP, 2 de assistolia e 1 de *torsades de pointes* . ^9^ Em nosso estudo, a incidência de parada cardiorrespiratória foi de 3,3%, também com predomínio de ritmos não chocáveis. A redução da ocorrência de casos de FV/TV em relação a dos estudos iniciais pode hipoteticamente ser atribuída a mudanças no tratamento da COVID-19, com menor utilização de drogas que podem prolongar o intervalo QT, além da evolução da curva de aprendizado dos profissionais de saúde com a doença. O predomínio de ritmos não chocáveis também pode ser atribuído ao acometimento sistêmico e à intensa resposta inflamatória presente nos casos graves de COVID-19.

Nosso estudo apresenta limitações que devem ser consideradas. O número de pacientes incluídos é relativamente pequeno, refletindo a experiência inicial de atendimento. Os pacientes internados em enfermaria não estavam em monitorização cardíaca contínua, assim, episódios de arritmia assintomáticos podem não ter sido relatados. O diagnóstico de arritmias foi obtido a partir da revisão dos registros de prontuário, sendo que em alguns casos a arritmia descrita foi visualizada apenas em monitor e não foi feito o registro em eletrocardiograma de 12 derivações. Não foram obtidos dados laboratoriais de marcadores de lesão e/ou disfunção miocárdica, como troponina e BNP, dados sobre formas de ventilação não invasiva, momento e doses no uso de drogas vasoativas, distúrbios hidroeletrolíticos e histórico prévio de arritmias, todos potencialmente associados à ocorrência de arritmias durante a internação. Trata-se de estudo com coleta de dados retrospectiva e em centro único terciário. Dessa forma, seus resultados não podem ser generalizados para outros cenários clínicos.

## Conclusões

Neste estudo de coorte de pacientes com COVID-19 internados em hospital de referência brasileiro, a incidência de arritmias cardíacas foi de 8,7%, sendo taquiarritmia atrial a mais comum. A presença de insuficiência cardíaca foi associada a maior risco de arritmias cardíacas. Pacientes com COVID-19 atendidos em parada cardiorrespiratória apresentam elevada mortalidade.
