# Supplementary material for: Arritmias Cardíacas em Pacientes com COVID-19
Source: Arq Bras Cardiol. 2021 Sep 3;117(5):1010–5. [Article in Portuguese] doi: 10.36660/abc.20200963 (PMC8682105; doi:10.36660/abc.20200963)
Supplement: Translation

# Translation: Cardiac Arrhythmias in Patients with COVID-19

**Authors:** Mauricio Pimentel, Ana Paula Arbo Magalhães, Camila Valvassori Novak, Bruna Miers May, Luiz Gustavo Bravosi da Rosa, Leandro Ioschpe Zimerman

**Affiliations:** 1 Porto Alegre RS Brazil Hospital de Clinicas de Porto Alegre, Porto Alegre, RS - Brazil; 2 Porto Alegre RS Brazil Programa de Pós-Graduação em Ciências da Saúde: Cardiologia e Ciências Cardiovasculares – Universidade Federal do Rio Grande do Sul, Porto Alegre, RS – Brazil

**Keywords:** COVID-19, Cardiac Arrhythmias, Heart Arrest, Atrial Fibrillation

## Abstract

**Background:**

The coronavirus disease 2019 (COVID-19) is associated with cardiovascular clinical manifestations, including cardiac arrhythmias.

**Objective:**

To assess the incidence of cardiac arrhythmias (atrial tachyarrhythmia, bradyarrhythmia, and sustained ventricular tachycardia) and cardiac arrest (CA) in a cohort of patients hospitalized with COVID-19 in a tertiary university-affiliated hospital.

**Methods:**

Cohort study with retrospective analysis of electronic medical records. For comparison between groups, a value of p <0.05 was considered statistically significant

**Results:**

We included 241 consecutive patients diagnosed with COVID-19 (mean age, 57.8 ± 15.0 years; 51.5% men; 80.5% white), 35.3% of whom received invasive mechanical ventilation (MV). The overall mortality was 26.6%, being 58.8% among those on MV. Cardiac arrhythmias were identified in 8.7% of the patients, the most common being atrial tachyarrhythmia (76.2%). Patients with arrhythmias had higher mortality (52.4% *versus* 24.1%, p = 0.005). On multivariate analysis, only the presence of heart failure (HF) was associated with a higher risk of arrhythmias (hazard ratio, 11.9; 95% CI: 3.6-39.5; p <0.001). During hospitalization, 3.3% of the patients experienced CA, with a predominance of non-shockable rhythms. All patients experiencing CA died during hospitalization.

**Conclusions:**

The incidence of cardiac arrhythmias in patients admitted with COVID-19 to a Brazilian tertiary hospital was 8.7%, and atrial tachyarrhythmia was the most common. Presence of HF was associated with an increased risk of arrhythmias. Patients with COVID-19 experiencing CA have high mortality.

## Introduction

The first cases of the disease caused by the severe acute respiratory syndrome coronavirus 2 (SARS-CoV-2), named COVID-19, were reported in China. Since then, COVID-19 has spread fast across the globe, being declared a pandemic by the World Health Organization on March 11, 2020. In Brazil, the number of confirmed cases of COVID-19 exceeded 3 million by August 2020. ^1^

In COVID-19 case series, cardiovascular complications, such as myocardial injury, arrhythmias, myocarditis, heart failure, and cardiogenic shock, have been reported. ^2^ The damage to the cardiovascular system might have a multifactorial cause, including direct viral myocardial injury, exaggerated systemic inflammatory response, and thromboembolic phenomena. ^3^ Viral action via the angiotensin-converting-enzyme 2 receptor and its down regulation effect are factors involved in the exaggerated inflammatory response. ^4^ In addition, regarding cardiac arrhythmias, the following possibilities can be considered: proarrhythmic effects of the drugs used for the treatment of COVID-19, hypoxia caused by viral lung involvement, myocardial ischemia, water-electrolyte imbalance, myocardial strain, and intravascular volume changes. ^5^ The unbalanced inflammatory response by type 1 and type 2 T helper cells is another mechanism proposed to explain inflammation and arrhythmogenesis in patients with COVID-19. ^6^ The first case series from China have shown an incidence of cardiac arrhythmias of 17%, which could reach 44% in patients admitted to the intensive care unit (ICU). ^7^ Those studies, however, have detailed neither the type nor the characteristics of the arrhythmias observed. More recent studies conducted in North American centers have reported an overall incidence of arrhythmias of 6% and of atrial tachyarrhythmias of 16%. ^8^^–^^10^

This study aimed to assess the incidence of cardiac arrest and cardiac arrhythmias in a cohort of patients with COVID-19 admitted to a Brazilian tertiary university-affiliated hospital.

## Methods

Cohort study including consecutive patients diagnosed with COVID-19 admitted to the Hospital de Clínicas de Porto Alegre, Rio Grande do Sul state, from March 1st to July 20, 2020. The first 241 consecutive patients diagnosed with SARS-CoV-2 infection confirmed by use of reverse transcription polymerase chain reaction of nasal and oropharyngeal swabs were assessed.

All electronic medical records were reviewed for the collection of demographic data and comorbidities, hospital outcome (death or hospital discharge), need for invasive mechanical ventilation (MV), and occurrence of cardiac arrest and cardiac arrhythmias. Medical and nursing data, as well as electrocardiographic findings, when available in the electronic medical record system, were analyzed.

Regarding the cases of cardiac arrest, the initial rhythm was reviewed and classified as: ventricular fibrillation/ventricular tachycardia (VF/VT), asystole, bradyarrhythmia, and pulseless electric activity (PEA). The occurrence of cardiac arrhythmias was defined as the presence of sustained atrial tachyarrhythmias (atrial fibrillation, atrial flutter), bradyarrhythmias, and sustained ventricular tachycardia. Arrhythmias present on hospital admission were not included, only those occurring during hospitalization. The study protocol was approved by the Committee on Ethics and Research of the Group of Research and Postgraduation of the Hospital de Clínicas de Porto Alegre.

### Statistical analysis

The continuous variables with normal distribution were described as mean and standard deviation. The length of hospital stay showed no normal distribution by the Shapiro-Wilk test, was presented as median and interquartile range, and compared by use of Mann-Whitney test. The demographic and clinical characteristics were compared between patients with and without cardiac arrhythmias by using nonpaired Student *t* test for continuous variables and chi-square test for categorical variables. The association between clinical variables and the occurrence of cardiac arrhythmias was assessed with univariate and Cox multivariate analysis models. A two-tailed p value < 0.05 was considered statistically significant. All analyses were performed using the SPSS software, version 14.0, for Windows.

## Results

This cohort study included 241 consecutive patients hospitalized with COVID-19 and mean age of 57.8 ± 15.0 years, 51.5% of whom were men, and 80.5% were white. The median length of hospital stay was 9 (interquartile range: 5-17) days, and 35.3% of the patients required MV. The length of hospital stay was longer in patients who had cardiac arrhythmias. The overall mortality was 26.6%, being 58.8% among those on MV and 9% among those not requiring MV (p=0.001).

Cardiac arrhythmias, defined as the presence of sustained atrial tachyarrhythmias, bradyarrhythmias and sustained ventricular tachycardia, were observed in 21 patients (8.7%). Table 1 shows the demographic and clinicals characteristics of the patients with and without arrhythmias. Of those with arrhythmias, 16 (76.2%) had sustained atrial tachyarrhythmias, 3 (9.5%) had sustained ventricular tachycardia, and 2 (9.5%) had bradyarrhythmias. Patients with arrhythmias had higher mortality, 52.4% *versus* 24.1% (p=0.005). Cardiac arrhythmias were more frequent in men, patients on MV, and those with history of heart failure. Table 2 shows the results of the univariate and Cox multivariate analysis for the occurrence of cardiac arrhythmias. In that model, only the presence of heart failure associated significantly with a higher risk for cardiac arrhythmias (hazard ratio: 11.9; 95% CI: 3.6-39.5; p<0.001). When adjusted for the presence of heart failure, the occurrence of cardiac arrhythmias was associated with a higher risk for total mortality (hazard ratio: 3.4; 95% CI: 1.8-6.7; p<0.05).

**Table 1:** Clinical characteristics of patients with and without cardiac arrhythmias

	All patients (n = 241)	With arrhythmia (n = 21)	Without arrhythmia (n = 220)	p value
Age, years	57.8 ± 15.0	62.6 ± 13.4	57.3 ± 15.0	0.11
Men	124 (51.5)	15 (72.4)	109 (49.5)	0.05
BMI, kg/m^2^	30.4 ± 6.3	29.3 ± 5.0	30.5 ± 6.4	0.43
White skin color	194 (80.5)	17 (81)	177 (80.5)	0.24
Mechanical ventilation	85 (35.3)	14 (66.7)	71 (32.2)	0.002
Hospital length of stay, days	9 (5-17)	25 (12-43)	9 (5-16)	0.001
Death	64 (26.6)	11 (52.4)	53 (24.1)	0.005
Comorbidities				
SAH	123 (51)	14 (66)	109 (49.5)	0.13
DM	64 (26.6)	7 (33.3)	57 (25.9)	0.46
HF	15 (6.2)	5 (23.8)	10 (4.5)	0.001
Pulmonary disease	52 (21.6)	7 (33.3)	45 (20.5)	0.17
Chronic kidney disease	29 (12)	-	29 (13.2)	0.07
Drugs				
Hydroxychloroquine	43 (17.8)	3 (14.3)	40 (18.2)	0.65
Anticoagulants	39 (16.2)	2 (9.5)	37 (16.8)	0.24

*Data were expressed as mean ± standard deviation or absolute numbers (percentage). The hospital length of stay was expressed as median and interquartile range. BMI: body mass index; SAH: systemic arterial hypertension; DM: diabetes mellitus; HF: heart failure* .

**Table 2:** Univariate and multivariate analysis for the outcome ‘cardiac arrhythmias’

	Univariate analysis			Multivariate analysis		
	HR	95% CI	p	HR	95% CI	p
Men	2.04	0.79-5.32	0.14	1.65	0.62-4.40	0.31
Mechanical ventilation	2.13	0.75-6.04	0.15	2.57	0.88-7.49	0.08
Heart failure	11.10	3.48-35.3	0.01	11.91	3.59-39.46	0.01

*HR: hazard ratio; CI: confidence interval.*

During hospitalization, 8 patients (3.3%), all of them admitted to the ICU, experienced cardiac arrest, and their clinical characteristics are shown in Table 3 . Figure 1 shows the distribution of the cardiac arrest rhythms: VF/VT, 2 patients (25%); PEA, 3 patients (37.5%); and asystole, 3 patients (37.5%). All patients undergoing cardiac arrest died during hospitalization.

**Table 3:** Characteristics of the cardiac arrests of patients with COVID-19

Patient number	CA on hospitalization day	CA rhythm	Clinical description	Outcome
1	1	PEA	26 years, asthma, obesity, and schizophrenia	ROSC 20 minutes, anoxic encephalopathy, comfort measures, death
2	26	PEA	54 years, renal transplant patient	ROSC 2 minutes, progressed to refractory shock, death
3	25	VF/VT	58 years, dilated cardiomyopathy, ICD, ARDS and MV	ROSC 20 minutes, refractory shock, death
5	10	VF/VT	45 years, dilated cardiomyopathy	ROSC 12 minutes, refractory shock, death
6	43	Asystole	71 years, ischemic heart disease	Death
7	12	Asystole	63 years, SAH, DM	Death
8	01	PEA	76 years, ischemic heart disease	Death
9	25	Asystole	41 years, SAH, obesity	ROSC 35 min, multiple organ dysfunction, death

*CA: cardiac arrest; PEA: pulseless electric activity; VF/VT: ventricular fibrillation/ventricular tachycardia; ROSC: return of spontaneous circulation; ICD: implantable cardioverter-defibrillator; ARDS: adult respiratory distress syndrome; SAH: systemic arterial hypertension; DM: diabetes mellitus; MV: mechanical ventilation.*

**Figure 1:** *Rhythms of cardiac arrest of patients with COVID-19. VF/VT: ventricular fibrillation/ventricular tachycardia; PEA: pulseless electric activity* .

## Discussion

In this cohort study including consecutive patients with COVID-19 admitted to a referral hospital, the overall mortality was 26.6%, and the incidence of arrhythmias, 8.7%, and of cardiac arrest, 3.3%. Atrial tachyarrhythmia was the most common arrhythmia, accounting for 76.2% of the arrhythmias. On multivariate analysis, the presence of heart failure was the only variable associated with a higher risk for cardiac arrhythmias. The length of hospital stay of patients with arrhythmias was longer than that of those without arrhythmia. This might be due to the need to treat the arrhythmia itself or represent the higher complexity and severity of patients developing arrhythmias.

The initial series have shown incidence of cardiac arrhythmias in patients with COVID-19 ranging from 7% to 17%, but have not specifically described their types. ^7^^,^^10^ In the New York State cohort, the incidence of arrhythmias was associated with different combinations of drugs used to treat COVID-19, and ranged from 10% to 20%, but the types of arrhythmia assessed were not defined. ^11^ In our study, we found no association of the use of hydroxychloroquine with higher risk for arrhythmias. The specific incidence of each type of arrhythmia has been described only recently. Data from an international registry including 1197 electrophysiology professionals have shown that atrial fibrillation was the most frequently described arrhythmia in patients with COVID-19. ^12^ A study with 115 patients has found an incidence of atrial tachyarrhythmias of 16.5%, which reached 27.5% in patients admitted to the ICU. ^8^ The largest study specific to arrhythmias published so far has assessed 700 patients hospitalized for 9 weeks. ^9^ During follow-up, 44 patients (6.3%) had cardiac arrhythmias, such as atrial fibrillation, bradyarrhythmias, and nonsustained ventricular tachycardia, and atrial fibrillation was the most frequent (57%). Presence of heart failure and ICU admission were significantly associated with a higher risk for arrhythmias. In that cohort, 11% of the patients were admitted to the ICU and overall mortality was 4%. Our incidence of arrhythmia of 8.7% can be considered close to that of that study, as well as the predominance of atrial arrhythmias and the association of heart failure with a higher risk of arrhythmia. However, our study included patients with more severe disease (35% required MV), and the presence of nonsustained ventricular tachycardia was not listed as an outcome. The use of MV was associated with a trend towards a higher risk for arrhythmia, although not statistically significant. The confirmation of the finding that patients with heart failure are at a higher risk for arrhythmias can indicate the need for more careful monitoring of such patients during hospitalization.

In the international survey with electrophysiology professionals, 4.8% reported cases of VF/VT and 5.6%, of PEA. ^12^ In the initial series from China, there was no specific mention to the occurrence of cardiac arrest and its rhythms. ^2^^,^^7^ In one of the studies, the incidence of VF/VT was 5.9%, and higher in patients with elevated troponin levels. ^2^ In the New York State cohort, the incidence of cardiac arrest ranged from 6% to 15%, depending on the different combinations of drugs used to treat COVID-19. ^11^ The rhythms of cardiac arrest were not described. In the study by Bhatla et al. described above, of the 9 cases of cardiac arrest (1.3%) reported, 6 were PEA, 2 were asystole, and 1, *torsades de pointes* . ^9^ In our study, the incidence of cardiac arrest was 3.3%, with predominance of non-shockable rhythms. The reduction in the occurrence of VF/VT cases as compared to that in the initial studies might be hypothetically attributed to changes in the COVID-19 treatment, with less use of drugs that can prolong the QT interval, in addition to the evolution of the learning curve of health professionals regarding the disease. Moreover, the predominance of non-shockable rhythms can be attributed to the systemic impairment and intense inflammatory response present in cases of severe COVID-19.

Our study has limitations that should be considered. The number of patients included is relatively small and reflects our initial experience. Patients admitted to the wards were not on continuous cardiac monitoring; thus, asymptomatic episodes of arrhythmia might not have been reported. The diagnosis of arrhythmia was obtained from review of medical records, and in some cases the arrhythmia described was only visualized on a monitor and no 12-lead electrocardiogram was recorded. The following data potentially associated with the occurrence of arrhythmias during hospitalization were not obtained: markers of myocardial injury and/or dysfunction, such as troponin and BNP; data on noninvasive ventilation modes; time of use and doses of vasoactive drugs; water-electrolyte imbalance; previous history of arrhythmias. This is a study with retrospective data collection in a single tertiary center. Therefore, our results cannot be generalized to other clinical settings.

## Conclusions

In this cohort study of patients with COVID-19 admitted to a Brazilian referral hospital, the incidence of cardiac arrhythmias was 8.7%, and atrial tachyarrhythmia was the most common. The presence of heart failure was associated with a higher risk of cardiac arrhythmias. Patients with COVID-19 and experiencing cardiac arrest have high mortality.
